# Identification of mycoparasitism-related genes in *Clonostachys rosea* 67-1 active against *Sclerotinia sclerotiorum*

**DOI:** 10.1038/srep18169

**Published:** 2015-12-14

**Authors:** Zhan-Bin Sun, Man-Hong Sun, Shi-Dong Li

**Affiliations:** 1 Institute of Plant Protection, Chinese Academy of Agricultural Sciences, Beijing 100193, China

## Abstract

*Clonostachys rosea* is a mycoparasite that has shown great potential in controlling various plant fungal pathogens. In order to find mycoparasitism-related genes in *C. rosea*, the transcriptome of the efficient isolate 67-1 in association with sclerotia of *Sclerotinia sclerotiorum* was sequenced and analysed. The results identified 26,351 unigenes with a mean length of 1,102 nucleotides, among which 18,525 were annotated in one or more databases of NR, KEGG, Swiss-Prot, GO and COG. Differentially expressed genes at 8 h, 24 h and 48 h after sclerotial induction were analysed, and 6,890 unigenes were upregulated compared with the control without sclerotia. 713, 1,008 and 1,929 genes were specifically upregulated expressed, while 1,646, 283 and 529 genes were specifically downregulated, respectively. Gene ontology terms analysis indicated that these genes were mainly involved in metabolism of biological process, catalysis of molecular function and cellular component. The expression levels of 12 genes that were upregulated after encountering with *S. sclerotiorum* were monitored using real-time PCR. The results indicated that the quantitative detection was consistent with the transcriptome analysis. The study provides transcriptional gene expression information on *C. rosea* parasitizing *S. sclerotiorum* and forms the basis for further investigation of mycoparasitism-related genes of *C. rosea*.

*Sclerotinia sclerotiorum* (Lib.) de Bary is a destructive fungal pathogen that infects more than 450 species of plants worldwide and causes huge economic losses in some important agricultural crops and vegetables, such as soybean, rape and carrot^1^. In most cases, *S. sclerotiorum* exists in infected plants, seeds and soil in the form of sclerotia which can survive for a long time. Under suitable temperature and humidity conditions, the sclerotia germinate and infect the leaves, stems and fruit, or infect plants directly through mycelium^2^,^3^. Therefore, the pathogen *S. sclerotiorum* is hard to eliminate.

The control of Sclerotinia diseases includes pesticides, agricultural practices and biopesticides, among which biocontrol has received more attention because of its environmental and food security advantages. *Clonostachys rosea* is a mycoparasite of various plant pathogenic fungi and has shown great potential to control numerous plant diseases^4^,^5^,^6^. However, the molecular mechanism of mycoparasitism of the fungus has not yet been clarified.

Mycoparasitism is an important method for biocontrol fungi against fungal pathogens. The mycoparasites attach to hyphae of the pathogens, suppress extension of germ tubes, and cause mycelial deformity. Tu^7^ observed the interaction of *Gliocladium virens* and *S. sclerotiorum* by using electron microscopy and revealed that the mycoparasite formed appressorium-like structures on the host fungus, penetrated and parasitized internal cells of sclerotia. Similar structures were also detected in *Trichoderma* spp. and *C. rosea* during their parasitic processes^8^,^9^. Up to now, several mycoparasitism-related genes have been identified and verified, many of which are cell wall degrading enzymes and were isolated from *Trichoderma* spp. The disruption of endochitinase^10^ and β-1,6-glucanase^11^ encoding genes in *T. virens* remarkably reduced the control efficiencies against pineapple and cotton diseases, while overexpression of the extracellular serine protease gene *tvsp1* increased the ability to protect cotton seedlings against *Rhizoctonia solani*^12^. Malmierca *et al.*^13^,^14^ isolated a cytochrome P450 monooxygenase encoding gene *tri4* and a terpene synthase gene *tri5* which were critical in producing trichothecene harzianum A from *T. arundinaceum*, and found that the mutants deleted for *tri4* and *tri5* led to a drastic reduction of antifungal activity against *R. solani* and *Botrytis cinerea*. Cloning and analysis of differently expressed genes in *Coniothyrium minitans* during the mycoparasitic process against *S. sclerotiorum* were conducted, which identified genes encoding β-1,3-glucanase^15^,^16^, and those associated with MAP kinase cascade^17^ and heat shock factors^18^.

Several mycoparasitism-related and biocontrol-related genes of *C. rosea* have also been reported. Kosawang *et al.*^19^ disrupted a zearalenone encoding gene, *zhd101*, from *C. rosea*, which led to the loss of zearalenone detoxification ability and a weak pathogenicity to the zearalenone-producing fungus *Fusarium graminearum*. Mouekouba *et al.*^20^ demonstrated that two plant resistance related factors, *WRKY* and *MAPK*, were upregulated in *C. rosea* during its interaction with *B. cinerea*, and were involved in the suppression of tomato grey mould by inducing plant resistance. Zou *et al.*^21^ indicated that mutants deficient for the subtilisin-like extracellular serine proteases gene *prC* attenuated virulence to nematodes. Some proteins associated with physiology and metabolism of the microorganism, e.g. zinc finger transcription factor and ATP-binding cassette transporter, were also confirmed to be involved in the parasitic process of the fungus against nematodes and pathogenic fungi^22^,^23^. However, the molecular mechanisms by which *C. rosea* parasitizes *S. sclerotiorum* are unclear. In previous study, a microscopic observation of sclerotia section of *S. sclerotiorum* treated by *C. rosea* 67-1 was performed, revealing that the hypha of the mycoparasite penetrated into the sclerotia and the tissue of the fungal host collapsed^24^. In this paper, the transcriptome of the isolate 67-1 parasitizing sclerotia was sequenced and analysed to find mycoparasitism-related genes. The results will provide a valuable foundation for further exploration of functional genes and molecular mechanism of *C. rosea* against *S. sclerotiorum*.

## Results

### De novo transcriptome assembly

The transcriptome of *C. rosea* 67-1 under sclerotial induction and on PDA medium was sequenced using the Illumina HiSeq 2000 platform. 181,076,576 raw reads were obtained from the six mycelia samples collected in different alimentation modes and stages. After filtering low quality reads and adaptor sequences, 14,468,003,640 bp of high quality clean reads remained and were further assembled. The Q_20_ values of the six samples were higher than 95.5%, and the GC contents were 53.7–55.6%. The clean data were assembled de novo using the Trinity software and 26,351 unigenes, comprising 29,036,163 bp, were obtained, including 6,526 distinct clusters and 19,825 distinct singletons, with a mean length of 1,102 bp and an N_50_ of 1,861 bp. The contigs and unigenes of different samples were analysed statistically, and only 1,779 (6.75%) unigenes were longer than 3,000 bp (Table 1).

### Functional annotation and classification of the 67-1 transcriptome

Functional annotation of all the unigenes was conducted, and a total of 18,525 unigenes (70.2%) could be annotated in one or more databases, among which 18,324 (98.9%), 11,371 (61.4%), 11,595 (62.6%), 8,615 (46.5%) and 8,808 (47.5%) were annotated to the NR, Swiss-Prot, KEGG, COG and GO databases, respectively, with an *E*-value < 10^−5^. The remaining 7,862 unigenes (29.8%) that lacked annotation information might represent new genes. Using Blast and ESTscan software, coding the sequences (CDSs) of 19,380 unigenes were successfully predicted.

**Figure 1:**
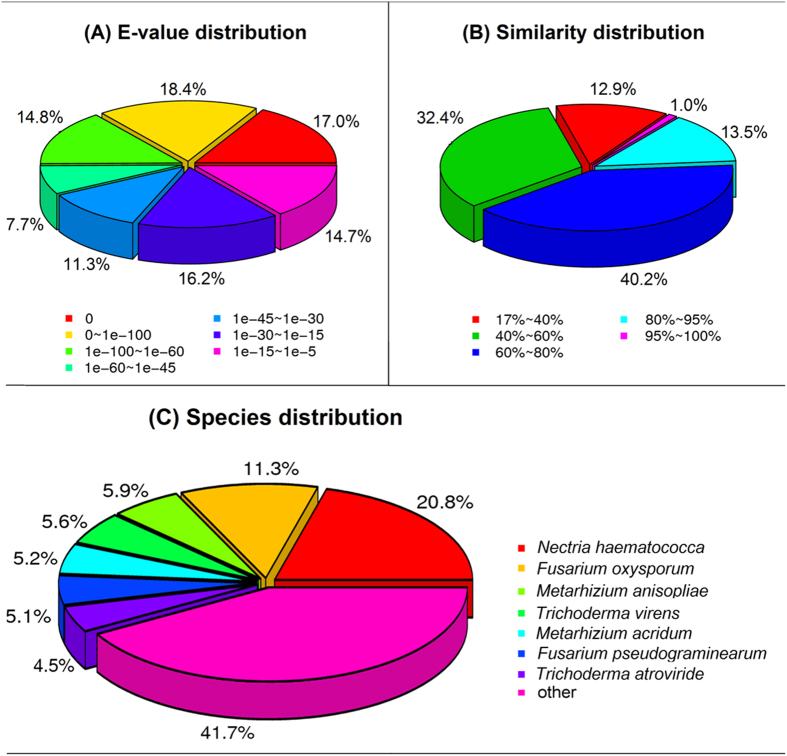
Non-redundant (NR) classification analysis of the unigenes derived from the transcriptome of *C. rosea* 67-1 grown on PDA and parasitizing on the sclerotia of *S. sclerotiorum*. (**A–C**) represent the *E*-value distribution, similarity distribution and species distribution of the NR annotation, respectively.

The distribution of *E*-values in the NR annotation indicated that 50.2% of the unigenes had a high homology, with *E*-value less than 10^−60^, and the similarity distribution indicated that 54.7% of the unigenes had a similarity higher than 60%. More than half of the annotated genes were related to parasitic fungi, plant pathogens or biocontrol fungi. 20.8% were associated with *Nectria haematococca* mpVI 77-13-4, a teleomorph of destructive plant pathogen *F. solani*; 11.3% were associated with *F. oxysporum* Fo5176; and 5.1% with *F. pseudograminearum* CS3096. A number of unigenes matched with genes from biocontrol fungi such as *Metarhizium anisopliae* ARSEF 23 (5.9%), *M. acridum* CQMa 102 (5.2%), *T. virens* Gv29-8 (5.6%) and *T. atroviride* IMI 206040 (4.5%) (Fig. 1).

**Figure 2:**
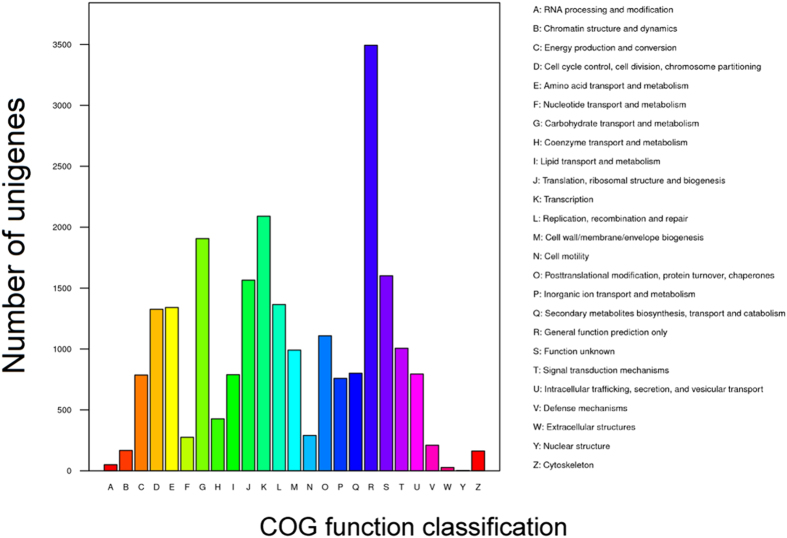
Clusters of orthologous groups (COG) functional classification of the unigenes of *C. rosea* 67-1. 8615 unigenes were functionally predicted and classified into 25 categories. Notations on the right represent the functions of the fungus.

The functions of 8,615 unigenes annotated in the COG database were predicted and classified into 25 categories. The largest group was ‘general function prediction only’, representing 40.5% of the COG-annotated unigenes. ‘Transcription’ was second, representing 24.2% of total unigenes, followed by the groups ‘carbohydrate transport and metabolism’ (22.1%), ‘function unknown’ (18.6%), and ‘translation, ribosomal structure and biogenesis’ (18.2%) (Fig. 2).

**Figure 3:**
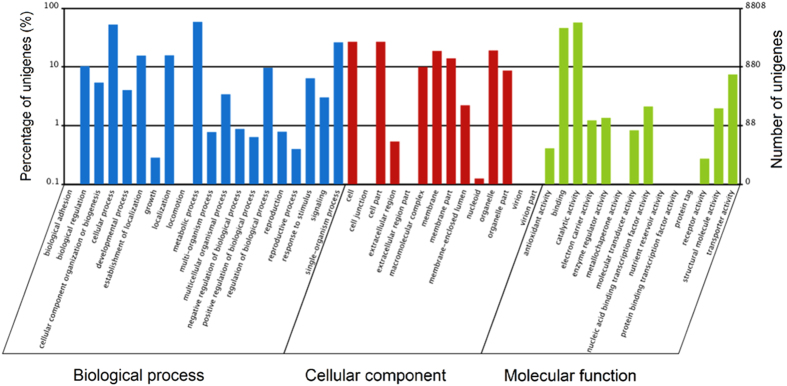
Gene ontology (GO) categories of the unigenes of *C. rosea* 67-1. Three kinds of function, biological process, cellular components and molecular functions, are included in the GO analysis, and the percentage and number of the unigenes in each category are shown separately.

The functions of the unigenes were further predicted using GO assignments, a standard system for gene function classification. Among its three ontologies, 18,895 unigenes matched 20 terms in the biological process group, 11,166 unigenes matched 14 terms in the cellular component group, and 10,455 unigenes matched 14 terms in the molecular function group. Most unigenes were associated with physiological and metabolic processes in the living organism; however, several genes might be related to its interaction with other fungi, though the number of these categories was much low. For example, seven unigenes were involved in ‘locomotion’ and ‘biological adhesion’. A number of unigenes with the features of ‘catalytic activity’ and ‘binding function’ might also play important roles in different nutrition modes of the biocontrol fungus (Fig. 3).

To analyse gene products associated with intracellular metabolic processes, the KEGG pathway database was used and 11,595 unigenes involved in 108 KEGG pathways were identified. The analysis indicated that ‘metabolic pathways’, ‘biosynthesis of secondary metabolites’ and ‘starch and sucrose metabolism’ were the dominant pathways active during *C. rosea* 67-1′s recognition and parasitization of *S. sclerotiorum*: the proportions reached 31.6%, 13.4% and 8.9% respectively. However, some pathways were associated with very few unigenes: ‘C5-branched dibasic acid metabolism’ (six), and ‘lipoic acid metabolism’ (three) and ‘caffeine metabolism’, (three) (Table 2).

### Identification of differentially expressed genes (DEGs) of *C. rosea* 67-1

**Figure 4:**
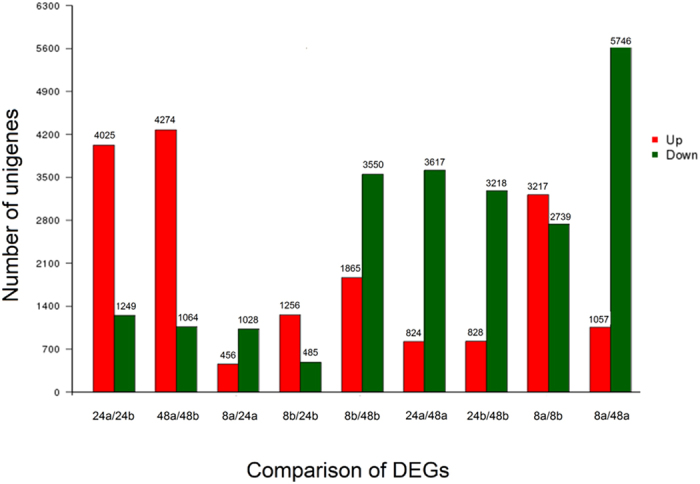
Differentially expressed genes (DEGs) in *C. rosea* 67-1 grown on PDA medium and under sclerotial induction at 8 h, 24 h and 48 h. Pairwise comparisons were performed between the fungus with different modes of nutrition. Small letters ‘a’ and ‘b’ represent isolate 67-1 grown on PDA medium and parasitizing on sclerotia, respectively. The numbers before the letters indicate the sampling time points (hours). The bars in red indicate the genes with upregulated expression, and the bars in green indicate genes with downregulated expression during the mycoparasitic process.

The gene expressions of 67-1 parasitizing on sclerotia and vegetative growth on PDA medium were compared at each time point and 10,504 DEGs were detected in the two growth conditions (Fig. 4). During the parasitic process, a total of 6,890 unigenes were upregulated, among which 604 rose continuously over the time course, and 713 DEGs were early stage-specific (0–8 h), 1,008 were middle stage-specific (8–24 h), and 1,929 were late stage-specific (24–48 h). Meanwhile 3,614 genes were found downregulated, including 282 continued falling genes and 1,646, 283 and 529 specific DEGs at 8 h, 24 h and 48 h, respectively. At the beginning of parasitism, numerous of unigenes changed their expression dynamically under sclerotial induction; 3,217 genes were upregulated and 2,739 were downregulated compared with the fungus grown on nutrient plates. With increasing time, the number of upregulated unigenes increased, while the downregulated unigenes decreased. At 48 h, gene expressions of the biocontrol fungus were relative stable. Almost all the DEGs with maximum variations (either upregulated or downregulated) in different nutrient modes and growth stages were unknown genes or hypothetical genes with no specific functions reported.

The analysis of GO category and functional enrichment of the DEGs under sclerotial induction and vegetative growth showed the same enrichment terms in each category at 8 h, 24 h and 48 h, indicating an ongoing process of mycoparasitism; however, the proportions of specific functions differed among sampling times. The terms catalytic activity, cell and cell part and organic substance metabolic process were the most relevant and significant GO classifications of molecular function, cellular component and biological process, respectively.

By KEGG pathway assessment, all the DEGs were assigned to five types, with 108 KEGG pathways. The most frequently represented type was ‘metabolic pathway’, mainly including ‘metabolic pathways’, ‘biosynthesis of secondary metabolites’ and ‘amino sugar and nucleotide sugar metabolism’. The second largest type was ‘genetic information processing’, mainly including ‘RNA transport’ and ‘protein processing in endoplasmic reticulum’. ‘Cell cycle-yeast’ and ‘meiosis-yeast’ in the category of ‘cellular process’ also acted as typical pathways. However, the pathways of ‘environmental information processing’ with the typical pathway of ‘MAPK signalling pathway’ and ‘organismal systems’, represented by ‘natural killer cell mediated cytotoxicity’, had a quite low frequencies compared with the other types of pathways.

### Quantitative real-time PCR verification

**Figure 5:**
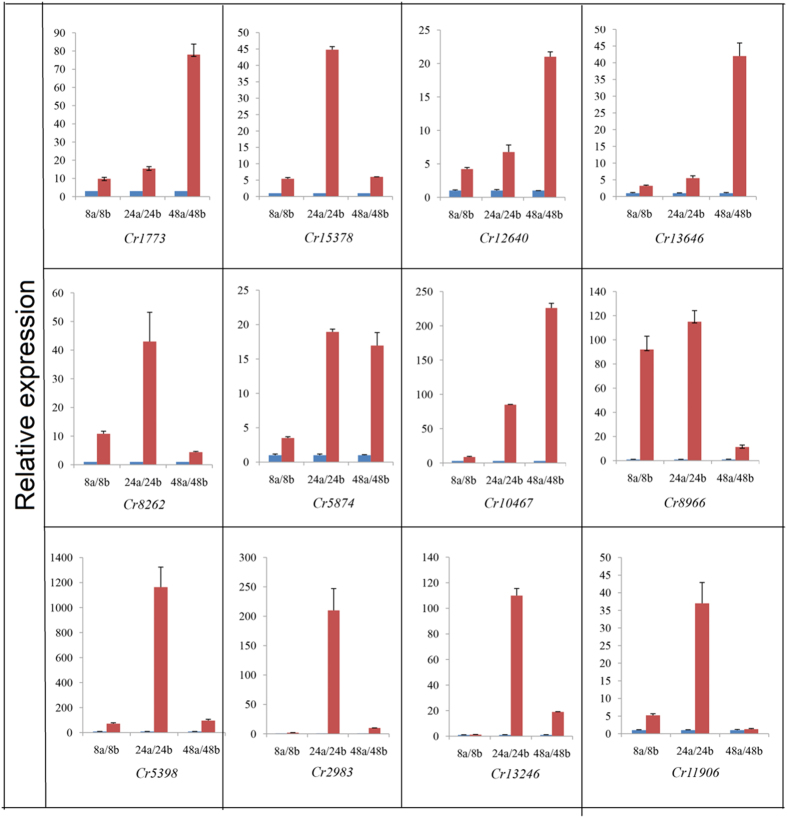
Verification of differentially expressed genes of *C. rosea* 67-1 under induction of sclerotia of *S. sclerotiorum* using quantitative real-time PCR. Relative expression levels of 12 unigenes (*Cr1773*, *Cr15378*, *Cr12640*, *Cr13646*, *Cr8262*, *Cr5874*, *Cr10467*, *Cr8966*, *Cr5398*, *Cr2983*, *Cr13246* and *Cr11906*) from 67-1 during vegetative growth and during the mycoparasitic process are compared pairwise at different sampling times. The bars in blue with the small letter ‘a’ represent the control, and the bars in red with the letter ‘b’ represent treatment by adding fresh sclerotia. Error bars indicate the standard deviation of three replicates.

Twelve DEGs were selected and divided into two groups according to their expression levels. In the first group, containing four genes encoding an MFS transporter, aldehyde dehydrogenase, carbonic anhydrase and cysteine synthase, the gene expression levels increased successively throughout the process of sclerotial induction. In the other group, the DEGs were upregulated at 24 h but decreased at 48 h. The genes encoding endochitinase, monooxygenase, sugar transporter, N amino acid transport system protein, endoribonuclease, 4-aminobutyrate aminotransferase, hypothetical protein and alpha-galactosidase fitted this pattern (Fig. 5). The results of real-time PCR quantification were consistent with the DEGs analysis of the 67-1 transcriptome, indicating that the data obtained from transcriptome analysis were reliable for further analysing and selecting of parasitism-related genes in *C. rosea* during sclerotial induction.

## Discussion

Understanding the mechanism of mycoparasitism of *C. rosea* would be of great value to improve biocontrol efficiency against plant fungal pathogens. In this study, a high-throughput sequencing and analysis of *C. rosea* induced by sclerotia of *S. sclerotiorum* was performed to explore mycoparasitism-related genes. The DEGs from the mycoparasite *T. harzianum* against fungal pathogens have been investigated by using suppression subtractive hybridization (SSH) method and RNA-seq technique in previous studies, in which autoclaved cell walls of *F. solani* and *S. sclerotiorum* were used as parasitic substrates compared with the saprophytic medium of glucose^25^,^26^. However, the inactive cell walls of pathogenic fungi were more likely to provide a nutrient for vegetative growth of the mycoparasites, although certain genes would be turned on and expressed during the process of decomposing and utilizing the special substance supplemented, e.g. the fungal cell wall or chitin. In our research, fresh, non-autoclaved sclerotia of *S. sclerotiorum* were added and acted as an inducer and substrate, which represented the status of *C. rosea* parasitizing on *S. sclerotiorum*. In order to ensure no *S. sclerotiorum* mixing up with *C. rosea* samples, we determined the germination of sclerotia on agar before transcriptome sequencing and analysis, and found no hyphae extending within sampling time. We amplified and sequenced ITS in the samples as well, which arose a single peak for each base, indicating that all the samples came from *C. rosea* (Data not shown). Several studies have suggested that when encountering with hyphae of pathogenic fungi, mycoparasites might display their biotrophic lifestyle^8^,^9^,^27^. Using live sclerotia as the substrate, more DEGs and more reliable candidates associated with mycoparasitism of *C. rosea* will be detected.

Generally, a series of activities are involved in the mycoparasitic process, including recognition, attachment, penetration and parasitism^28^, and these behaviours of the antagonist are triggered in different stages of parasitism. In the co-culture of 67-1 and sclerotia, we found that the hyphae of 67-1 extended and attached to the sclerotia in 8 h. After 48 h the surface of the sclerotia was covered with hyphae of the mycoparasite and the mycelium of *C. rosea* penetrating into internal tissues of the sclerotia was detected in the section under a microscope (Data not shown). Therefore, three time points (8 h, 24 h and 48 h), with and without sclerotia, were used to represent the whole mycoparasitic process of before penetration, mass invasion, and the later stage of mycoparasitism to construct the transcriptome of 67-1 parasitizing on sclerotia of *S. sclerotiorum*. The results showed that 3,217 unigenes were upregulated and 2,739 were downregulated at 8 h. We deduced that upon the addition of sclerotia, the *C. rosea* isolate changed its alimentation mode from saprophytic on PDA to parasitic, such that the fungus induced numerous genes related to parasitism and repressed many genes participating in vegetative growth on PDA medium. With increasing time, the upregulated unigenes increased successively compared with those expressed in the absence of sclerotia, indicating that the parasitic process of *C. rosea* was ongoing. However, the number of downregulated unigenes decreased compared with that at 8 h. We hypothesized that the fungus turned off many genes involved in normal vegetative growth throughout the sampling times, resulting the observed decrease in downregulated unigenes at 24 and 48 h compared with the control. However, these speculations need further verified.

According to species distribution analysis, more than half of the unigenes annotated were parasitism-related, including both plant fungal pathogens, e.g. *F. solani* and *F. oxysporum*, and entomogenous fungi *Metarhizium* spp. and mycoparasites *T. virens*, *T. atroviride*, which show strong parasitic abilities to their hosts. Common genes discovered in different species may play critical roles in certain functions. Analysing the unigenes expressed in *C. rosea* and detecting their expressions in other parasitic fungi represents an efficient and simple way to further identify and explore mycoparasitism-related genes.

Functional genes participating in the mycoparasitic process have received much attention in the last decades. Two genes encoding signal transduction proteins, G-protein and mitogen activated protein kinase (MAPK), were extensively studied and confirmed to be substantially involved in recognition and attachment of mycoparasites^29^−^31^. From the transcriptome of *C. rosea* 67-1, we also identified two unigenes encoding the G-protein alpha subunit and MAPK, respectively, whose expressions were remarkably upregulated under sclerotial induction compared with the control, indicating that both signal transduction-related proteins might be involved in regulating the activities of key enzymes in mycoparasitism or the formation of parasitic structures. Cell wall degrading enzymes are important factors in penetration and mycoparasitism^32^−^34^. When 67-1 was induced by sclerotia, a series of cell wall degrading enzymes encoding genes, such as endochitinase, endoglucanase and serine protease, were remarkably upregulated. These hydrolases might play important roles in the interaction between mycoparasites and fungal pathogens by degrading cell wall, involving in penetration, and suppressing spore germination and germ tube extension of the pathogens. From the transcriptome, we also obtained several toxin encoding genes, which might effect the physiological activities of the pathogen and inhibit mycelial growth, leading to an increased mycoparasitic activity of *C. rosea*.

As a mycoparasite, *C. rosea* attacks its host actively and directly; however, it also possesses a resistance response to toxic metabolites secreted by *S. sclerotiorum*. It was reported that ATP-binding cassette (ABC) transporter from *C. rosea* was able to resist antifungal materials produced by pathogens. An ABC transporter-encoding gene, *abcG5*, was isolated from the strain IK726 and functionally verified by gene deletion and complementation, which indicated that *abcG5* was involved in cell protection by detoxifying *Fusarium* mycotoxin zearalenone and other fungicides, thereby enhancing xenobiotic tolerance^19^,^22^. In our study, the expression of a gene encoding an ABC transporter was induced in *C. rosea* 67-1 in the presence of live sclerotia; thus, the toxin of *S. sclerotiorum* might stimulate the resistance system of *C. rosea*, and induce the expression of the ABC transporter-encoding gene to detoxify and protect *C. rosea*.

Among the DEGs of 67-1, a number of functional unknown or hypothetical genes were identified. These genes were dramatically upregulated under sclerotial induction, some of them by more than 1,000-fold. These genes, especially the DEGs not expressed or rarely expressed in nutrient agar, but highly expressed under sclerotial induction, might be significant in mycoparasitism of *C. rosea*. In the future, based on the information from the *C. rosea* genome^35^ and the parasitic transcriptome, we will screen target genes and verify their specific functions in mycoparasitism by gene deletion, complementation and bioassay of *S. sclerotiorum in vitro* and in the greenhouse.

## Methods

### Strains

*C. rosea* 67-1 was originally isolated from a vegetable yard in Hainan Province, China. *S. sclerotiorum* Ss-H was obtained from infected stems of soybeans in a field in Heilongjiang Province. Both strains are maintained in potato dextrose agar (PDA) in the Biocontrol of Soilborne Diseases Laboratory of the Institute of Plant Protection, Chinese Academy of Agricultural Sciences.

### Preparation of sclerotia

Agar blocks of Ss-H were inoculated into carrot medium^36^ and incubated at 26 °C. After 15 days, the sclerotia were harvested by rinsing with tap water to remove remnants of the medium. The sclerotia were air dried and stored in a cool place.

### Preparation of mycelia samples of 67-1

The strain 67-1 was incubated on PDA plate at 26 °C for 10 days, and the spores were eluted by 5 ml sterile distilled water with a sterile glass spatula. The number of spores was counted under a microscope using a haemocytometer, and the concentration of the suspension was adjusted to 1 × 10^7^ spores/ml. 100 μl of 67-1 suspension was dropped on the centre of a PDA plate (ϕ 90 mm) covered with a 85-mm sterile cellophane and smeared evenly with a sterile glass scraper. The fungus was cultured in an incubator at 26 °C for 48 h.

Uniformly sized sclerotia were picked up and surface-sterilized in 2.5% sodium hypochlorite solution for 3 min, and rinsed with sterile distilled water five times. Excess water remaining on the sclerotia was blotted off using sterile filter paper. The sclerotia were put onto the surface of 67-1 plates evenly to cover the whole colonies and co-cultured consecutively in an incubator at 26 °C. The sclerotia on the plates were took out using sterile forceps at the early stage (8 h), the intermediate stage (24 h) and the later period (48 h) of mycoparasitism, respectively, and the mycelia of 67-1 growing on the cellophane were collected using a sterile spatula. The 67-1 plates without added sclerotia acted as the control. The six samples of fungal hyphae were frozen immediately in liquid nitrogen and kept at −80 °C. Five replicates were conducted for each treatment, and the experiment was repeated for three times.

### Construction of cDNA libraries and sequencing

The total RNAs of the mycelia samples of 67-1 were extracted using the Trizol reagent (Invitrogen, Carlsbad, CA, USA) according to the manufacturer’s protocol, and DNase I (Invitrogen) was used to digest the genomic DNA completely. The concentration of total RNA was determined using an Ultraviolet Spectrophotometer (NanoDrop ND-1000, Wilmington, DE, USA) and its quality was tested using an Agilent 2100 Bioanalyzer (Agilent Technologies, Santa Clara, CA, USA) and the NanoDrop ND-1000 using the standards of 28S/18S > 1.0, OD_260/230_ ≥ 1.8, OD_260/280_ ≥ 1.8, and RNA Integrity Number > 6.5. 67-1 mRNA was extracted with Oligo (dT) magnetic beads and broken into small pieces using fragmentation buffer. Short cDNA fragments were synthesized using random hexamer primers, purified, resolved with EB buffer for end reparation and connected with adapters. Suitable fragments were selected as templates for PCR amplification. The quality of the six cDNA libraries was monitored with an Agilent 2100 Bioanalyzer and StepOnePlus Real-Time PCR System (ABI, CA, USA), and their sequences were determined using an Illumina HiSeq™ 2000 (Illumina Inc., CA, USA) in the Beijing Genome Institute (BGI).

### Bioinformatic analysis of 67-1 transcriptome sequences

Raw reads of 67-1 cDNA libraries were filtered to remove adaptor sequences, low quality reads and unknown nucleotides larger than 5%, and the clean reads obtained were submitted to the NCBI sequence read archive (SRA). De novo transcriptome assembly was carried out using the short reads assembly program Trinity^37^. The high quality reads were assembled into contigs, and then integrated to obtain unigenes. All the samples were derived from the same strain of *C. rosea*; therefore, the unigenes in each assembly could be further processed by sequence splicing and redundancy removal to obtain longer non-redundant unigenes.

Blastx alignment (*E*-value < 10^−5^) was conducted to determine the sequence direction and to predict the protein coding regions. The CDSs were extracted from the unigenes, and those sequences that did not match any Blast results were predicted using the ESTScan program^38^. Annotation of the unigenes was performed using NR (Non-redundant), Swiss-Prot, GO (Gene ontology), KEGG (Kyoto encyclopedia of genes and genomes) and COG (Clusters of orthologous groups of proteins) databases. The program Blast2GO was used to obtain GO annotations regarding cellular component, biological process and molecular function of the unigenes^39^, and the Blastall software was used to predict and classify the COG and KEGG pathway-associated unigenes^40^,^41^.

### Analysis of DEGs

The expression levels of the 67-1 unigenes differentially expressed in different sampling times under sclerotial induction and on PDA medium were calculated by the fragments per kb per million fragments method (FPKM)^42^. The false discovery rate (FDR) is considered as a standard to confirm the threshold of *P* value in multiple tests and analyses^43^. An FDR < 0.001 and an absolute value ratio > 2 were adopted to judge the significance of gene expression differences between sclerotial induced and non-induced samples.

### Quantitative real-time PCR certification

To verify the reliability of the transcriptome of *C. rosea* 67-1, 12 genes that were upregulated in the DEG analysis were selected and quantified for their expression levels under sclerotial induction and during vegetative growth on PDA medium, using real-time PCR and internal reference gene *Elongation factor 1*^44^. Primer pair sets for the genes were designed using the software Primer Premier 6.0 (Table 3). The specificity of the primers was certified by conventional PCR with the following program: 94 °C for 3 min; 30 cycles of 94 °C for 1 min, 55 °C for 30 s and 72 °C for 30 s; followed by 72 °C for 10 min.

The total RNAs of the six samples were extracted and first strand cDNA was synthesized using a cDNA Synthesis Kit (Takara, Dalian, China). The expressions of the DEGs were assayed using a SYBR Premix Ex Taq (Takara) in an IQ 5^TM^ multicolor real-time PCR detection system (Bio-Rad, CA, USA) in a 25 μl reaction system containing 12.5 μl of SYBR Premix, 2 μl of 10-fold diluted cDNA, 1 μl of each primer and 8.5 μl of RNase-free water. The quantitative PCR was performed in a 96-well plate for 95 °C 2 min; 40 cycles of 95 °C 10 s and 55 °C 30 s. After the reaction, fluorescence values were collected every 0.5 °C from 55 °C to 85 °C for 81 cycles to check for non-specific amplification. The relative expression levels of the DEGs were calculated using the 2^–ΔΔCt^ method^45^. Three replicates were performed for each template at each time point.

### Statistical Analysis

The statistical software SAS 9.1.3 (SAS Institute Inc., Cary, NC, USA) was used for the analysis of variance (ANOVA) of the gene expression levels. Duncan’s multiple range test was used to compare the means of each treatment, and *P* value < 0.05 was considered significant.

## Additional Information

**How to cite this article**: Sun, Z.-B. *et al.* Identification of mycoparasitism-related genes in *Clonostachys* *rosea* 67-1 active against *Sclerotinia sclerotiorum*. *Sci. Rep.* **5**, 18169; doi: 10.1038/srep18169 (2015).

## Figures and Tables

**Table 1:** Statistics of the assembly quality of the transcriptome of *C. rosea* 67-1.

	Sample^1^	Total number	Total length	N_50_	TCS^2^	DC^3^	DS^4^
Contig	8a	48,302	20,160,931	974	—	—	—
	8b	46,601	20,726,522	1079	—	—	—
	24a	46,550	19,010,037	932	—	—	—
	24b	47,530	21,894,686	1191	—	—	—
	48a	39,178	16,046,611	958	—	—	—
	48b	39,245	19,122,207	1283	—	—	—
Unigene	8a	30,247	20,470,590	1105	30,247	3,980	26,267
	8b	30,165	20,243,658	1084	30,165	3,025	27,140
	24a	29,111	19,261,908	1063	29,111	3,730	25,381
	24b	29,521	21,923,337	1248	29,521	3,555	25,966
	48a	25,810	16,243,591	1069	25,810	2,790	23,020
	48b	26,270	19,603,112	1323	26,270	2,974	23,296
Total		26,351	29,036,163	1861	26,351	6,526	19,825

^1^Small letters ‘a’ and ‘b’ represent the isolate 67-1 growing on PDA medium and under sclerotial induction, respectively, and the numbers before the letters indicate the sampling times (hours).

^2^Total consensus sequence represents all unigenes assembled.

^3^Distinct cluster represents the cluster unigenes; the same cluster contains some highly similar (more than 70%) unigenes, which may come from same gene or a homologous gene.

^4^Distinct singleton represents the unigenes derived from a single gene.

**Table 2:** KEGG analysis of the transcriptome of *C. rosea* 67-1.

	Pathway	Proportion^1^ (%)	Level 1	Level 2
1	Metabolic pathways	31.6	Metabolism	Global map
2	Biosynthesis of secondary metabolites	13.4	Metabolism	Global map
3	Starch and sucrose metabolism	8.9	Metabolism	Carbohydrate metabolism
4	Amino sugar and nucleotide sugar metabolism	5.0	Metabolism	Carbohydrate metabolism
5	RNA transport	4.1	Genetic information processing	Translation
6	MAPK signaling pathway	4.1	Environmental information processing	Signal transduction
7	Purine metabolism	3.4	Metabolism	Nucleotide metabolism
8	Protein processing in endoplasmic reticulum	3.1	Genetic information processing	Folding, sorting and degradation
9	Cell cycle	2.9	Cellular processes	Cell growth and death
10	Spliceosome	2.5	Genetic information processing	Transcription
11	Meiosis	2.4	Cellular processes	Cell growth and death
12	RNA degradation	2.4	Genetic information processing	Folding, sorting and degradation
13	Pyrimidine metabolism	2.4	Metabolism	Nucleotide metabolism
14	Ribosome	2.1	Genetic information processing	Translation
15	Tyrosine metabolism	2.1	Metabolism	Amino acid metabolism
16	Lysine degradation	2.1	Metabolism	Amino acid metabolism
17	Peroxisome	2.0	Cellular processes	Transport and catabolism

The KEGG Pathway analysis is divided into three levels.

^1^Percentage of unigenes in a pathway from among all the genes with KEGG pathway annotation (11595). Proportions lower than 2% are not listed.

**Table 3:** Primers used in this study.

Gene	Predict function	Primers (5′-3′)
*Cr1773*	MFS transporter	F: CGCTACGGAGTATAATGACG
		R: TGTCTGCTTTGAACCCAC
*Cr5874*	Endoribonuclease	F: TGATGATATTCCCCGTCT
		R: TAGTCTCCAGGTTTCTCC
*Cr8262*	Monooxygenase	F: CGTTCCTGGCTAACTTGC
		R: TCCACCCTATCATCACCTC
*Cr10467*	Cysteine synthase	F: GAAGATGGACGGACGAAT
		R: GAAGGCTAAACAAGGAAGAA
*Cr12640*	Aldehyde dehydrogenase	F: GTTCACCCAAATGCTTCC
		R: CGATGATTCCGCAATAGTT
*Cr13246*	Alpha-galactosidase	F: GCTAATTGCACCACATCG
		R: CTAATACGCCAGAAAGGAA
*Cr15378*	N amino acid transport system protein	F: CATCACCATCGGAGTTGTA
		R: GAAGGAGTCGGAGGAAAA
*Cr5398*	Hypothetical protein	F: TTCACCCGCCAATGTATC
		R: CGTCCGCCAGAAGTATGT
*Cr13646*	Carbonic anhydrase	F: TGGATGACCAGCAAACTA
		R: CCAAATGAACGGGACAAT
*Cr8966*	4-aminobutyrate aminotransferase	F: GAATATCTGCGTGGTGCT
		R: TGAAGAAGGCCAAGGAGT
*Cr2983*	Sugar transporter	F: TTCTTTGCCGTGTTCTGT
		R: TCCTTCTTCGCTGTATCG
*Cr11906*	Endochitinase	F: GTTGTGGTTTGCCTGGTG
		R: CCGATACTCTGCTGCTCAT
